# Methyl-binding domain protein-based DNA isolation from human blood serum combines DNA analyses and serum-autoantibody testing

**DOI:** 10.1186/1472-6890-11-11

**Published:** 2011-09-06

**Authors:** Matthias Wielscher, Walter Pulverer, Johannes Peham, Manuela Hofner, Christine F Rappaport, Christian Singer, Christof Jungbauer, Christa Nöhammer, Andreas Weinhäusel

**Affiliations:** 1 Molecular Medicine, Austrian Institute of Technology, Muthgasse 11, 1190 Vienna, Austria; 2 Department of Obstetrics and Gynecology, Medical University of Vienna, Währinger Grütel 18 - 20, 1090 Vienna, Austria; 3 Blood Donation Center for Vienna, Lower Austria and Burgenland, Austrian Red Cross, Wiedner Hauptstraße 32, 1040 Vienna, Austria

## Abstract

**Background:**

Circulating cell free DNA in serum as well as serum-autoantibodies and the serum proteome have great potential to contribute to early cancer diagnostics via non invasive blood tests. However, most DNA preparation protocols destroy the protein fraction and therefore do not allow subsequent protein analyses. In this study a novel approach based on methyl binding domain protein (MBD) is described to overcome the technical difficulties of combining DNA and protein analysis out of one single serum sample.

**Methods:**

Serum or plasma samples from 98 control individuals and 54 breast cancer patients were evaluated upon silica membrane- or MBD affinity-based DNA isolation via qPCR targeting potential DNA methylation markers as well as by protein-microarrays for tumor-autoantibody testing.

**Results:**

In control individuals, an average DNA level of 22.8 ± 25.7 ng/ml was detected applying the silica membrane based protocol and 8.5 ± 7.5 ng/ml using the MBD-approach, both values strongly dependent on the serum sample preparation methods used. In contrast to malignant and benign tumor serum samples, cell free DNA concentrations were significantly elevated in sera of metastasizing breast cancer patients. Technical evaluation revealed that serum upon MBD-based DNA isolation is suitable for protein-array analyses when data are consistent to untreated serum samples.

**Conclusion:**

MBD affinity purification allows DNA isolations under native conditions retaining the protein function, thus for example enabling combined analyses of DNA methylation and autoantigene-profiles from the same serum sample and thereby improving minimal invasive diagnostics.

## Background

The prognostic potential of cell free nucleic acids in plasma and serum caused a lot of attention in current efforts to improve diagnoses of several cancerous diseases [1,2]. During the last years various approaches in this field, like the determination of genetic aberrations, the methylation status of the DNA [3], DNA level [4] and DNA-integrity [5] as well as miscellaneous proteomic approaches [6] have been reported to have clinical relevance. Despite this, breast cancer diagnosis still relies on physical examinations and mammography, because no accurate and reliable approach is currently available to be implemented into an effective diagnostic test. Therefore, the need for a simple non invasive blood test to perform routine pre-symptomatic screenings that improve early diagnosis is high.

Serum markers for breast cancer detection may be divided into two areas. On the one hand tumor-induced DNA aberrations detectable in serum of cancer patients which has been already examined in clinical trials [7]. On the other hand, there is the field of tumor-specific changes in proteins where serum-protein analyses [8] and the novel approach of tumor-autoantibody signatures [9,10] are applied for diagnostics. A combined analysis of cell free serum DNA and for example serum-autoantibodies should have the potential to increase the reliability and diagnostic power of non-invasive blood tests.

Due to the different demands on sample preparation and the very often limited sample material, a combined analysis of DNA and proteins for biomarker identification remains difficult. The currently often used silica membrane-based DNA isolation strategy does not allow subsequent protein analyses, because it relies mostly on protein-denaturation and includes standard ProteinaseK treatment of the samples [11,12].

To overcome these constraints, we developed a DNA isolation protocol based on the usage of a recombinantly expressed methyl binding protein (MBD) to extract genomic DNA from serum or plasma samples, thereby keeping the proteome intact.

MBD is the methyl CpG binding domain of the MeCP2 protein, which binds symmetrically methylated CpGs in any sequence context, and is involved in mediating methylation dependent transcriptional repression [13,14]. Although there is a strong evidence that MeCP2 binds exclusively methylated DNA fragments in vivo, a DNA methylation-independent binding activity of MeCP2 in vitro was also described in concordant literature [15,16], which makes it suitable for general in vitro DNA analysis.

Up to now, recombinant MBD protein, which is available upon overexpression of the cloned His-tagged protein in E.coli [17], has been predominantly used for DNA methylation analyses. The MBD protein has been preferably applied being immobilized in an affinity chromatography like manner with NaCl gradient elution steps to isolate methylated DNA for PCR and gel analyses [18], as well as for methylome profiling via Genomic Sequencing [19].

Alternative applications comprised DNA isolation from stool, where for example Zou H. et al applied a MBD column to minimize the background E. coli DNA [20], or direct binding of MBD to immobilized methylated DNA [21]. Beside these exclusively affinity based MBD applications, an assay was developed, where MBD was combined with methylation-sensitive restriction enzymes (COMPARE-MS) to minimize false positives for methylation assessment [22].

The workflow presented here uses MBD protein attached to Ni-Sepharose beads for affinity based-DNA purification that enables the simultaneous analyses of cell free serum DNA and serum proteins, resulting in a time and sample saving procedure. MBD isolated serum DNA has been found particularly suitable for DNA methylation analyses, and has allowed us to confirm an elevated level of cell free serum DNA of breast cancer patients with a malignant metastasizing neoplasm [23]. Autoantibody signatures of the protein fraction of MBD processed serum samples of control individuals were comparable to signatures derived from unprocessed serum of these individuals, indicating the suitability of MBD processed serum samples for immunological analyses upon DNA isolation.

## Methods

### Serum and plasma sample preparation

Serum and plasma samples were obtained from three different sources. Numbers of samples as well as type of processing are also given in Table 1. Serum samples from the Austrian Institute of Technology (source 1), age and sex matched, mean age of 27, taken from healthy volunteers, were prepared as follows: incubation of blood in Vacuette 9 ml Z Serum Clot Activator (Greiner Bio One, Frickenhausen, Germany) for 30 min followed by centrifugation at 1,800 × g for 10 min at room temperature. Serum-aliquots of 2 ml were stored at -80°C. The blood plasma was taken from the same donors as the serum at a single withdrawal. For the isolation of plasma, the blood samples were taken with a BD Vacutainer (BD diagnostics) glass whole blood tube with K3EDTA, centrifuged at 1300 × g for 10 min at 4°C and stored in 500 μl aliquots at -80°C (source 1).

**Table 1:** Overview of sample processing

Parameter	source 1	source 2	source 3
Origin	AIT	Austrian Red Cross	AKH
Sample type	serum/plasma	serum	serum
Test persons	control individuals age and sex matched	control individuals age and sex matched	control/breast cancer
Quantity	serum, n = 12 plasma, n = 8	serum, n = 48	non-canc., n = 30 cancer met., n = 12 cancer mal., n = 30 cancer ben., n = 12
Isolation	MBD/silica*	MBD/silica*	MBD/silica**
Analyses	DNA quantification multiplexed PCR test qPCR measurments array based autoanitbody tests	DNA quantification multiplexed PCR test	DNA quantification

AIT, Austrian Institute of Technology; AKH, Vienna General Hospital; cancer met., serum samples from cancer patients with a metastasizing tumor; cancer mal., patients with an malign tumor; cancer ben., derived from patient with a benign tumor; *DNA of all samples isolated by both isolation methods; ** DNA from 72 samples of this group was isolated using "silica", 12 using "MBD" MBD, methyl-binding domain protein; silica, silica membrane-based isolation

Serum samples provided by the Austrian Red Cross Blood Center (source 2) were treated according to the protocol described above. These serum samples were arranged into three sex matched subgroups: group 1 (36-45 years old), group 2 (46-55 years old), group 3 (56-65 years old). These samples were qualified as blood donors according the eligibility criteria of the Austrian Red Cross and are denoted as control individuals.

Samples obtained from the General Hospital in Vienna (AKH) consisting of control individuals, breast cancer patients and patients with metastasizing breast cancer were centrifuged at 1000 × g for 15 min and stored in 200 μl aliquots at -80°C until usage (source 3). The non-cancer patients of the AKH were defined upon personal interviews and examinations to exclude any familial history of breast cancer, any papable breast-nodules and any history or presence of cancerous disease.

Both the Red Cross Blood Center and the AKH provided anonymized samples after completion of all testing procedures and according to the institute's guidelines. The study was approved by the ethics committee of the Medical University Vienna and was carried out in compliance with the Helsinki Declaration.

### Protein expression and MBD-bead assembly

The E. coli strain BL21, containing the pET6HMBD plasmid, kindly provided by Sally H. Cross [24], was grown in Luria-Bertani medium with 30 μg/ml chloramphenicol and 50 μg/ml ampicillin. LB medium (10 ml) was inoculated with glycerol stock of E.coli strain BL21. Bacteria were grown for 8 h at 37°C and then further cultured over night in 250 ml LB medium. Culture was then split into three main cultures of 750 ml LB medium each for further culturing. The main cultures were grown in 2 l flasks for approximately 8 h, until an optical density (600 nm) of 0.6 was reached. Then recombinant protein expression was induced by adding Isopropyl-ß-D-thiogalactopyranosid (IPTG) to a final concentration of 0.4 mM. After incubation at 37°C over night, the E. coli biomass was collected by centrifugation at 4,000 rpm for 20 min. The E. coli biomass (approximately 3 g/l LB medium) was washed twice with PBS and resuspended in lyses buffer (20 mM HEPES, 1 M NaCl, 2 M Urea, 10% glycerol, 0.5 mM EDTA, 0.1% TritonX, pH = 8) to reach a concentration of 0.3 g biomass per ml. Samples were stored at -20°C until usage.

For MBD protein purification E.coli cells (800 μl) were mixed with 500 μl lyses buffer and lysed by repeated bead whirling mixing for 30 s in Lyses Matrix A tubes (MP Biomedicals, Eschwege, Germany) on a FastPrep24 instrument (MP Biomedicals). The bacterial extract was then centrifuged at 13,000 rpm for 10 min and the supernatant transferred to a new vial. Centrifugation at 13,000 rpm for 10 min was repeated until all cell debris was spun down.

A volume of 200 μl of a 50% suspension of Ni-Sepharose beads (Adar Biotech, Rehovot, Israel) were washed in 500 μl water, followed by centrifugation for 1 min at 1000 × g and removal of the supernatant from the beads. The beads were then equilibrated by adding 500 μl buffer A (20 mM HEPES, 100 mM NaCl, 10% glycerol, 20 mM beta-Mercaptoethanol (added fresh daily), 0.5 mM PMSF, 0.1 mM TritonX, pH = 8) and centrifuged at 1000 × g for 1 min. This procedure was repeated twice and then the beads were resuspended in 100 μl buffer A. His-tagged MBD-protein was bound to the equilibrated beads by addition of 800 μl bacterial extract (derived from approximately 250 mg E.coli wet cell weight) and 500 μl 2 × buffer A, and incubated for two hours on ice at which the beads were held in suspension upon repeated mixing.

MBD-loaded beads (200 μl) were washed twice by adding 500 μl wash buffer (buffer A, plus 10 mM Imidazol) followed by centrifugation at 1,000 × g for 1 min. The MBD protein was either eluted from beads with buffer A plus 500 mM Imidazol or used for DNA isolation. Protein concentration was measured with a BioRad Dc-Protein assay (Bio-Rad laboratories). Protein purification was visualized on a SDS page gel (NuPage Novex Bis-Tris Gel, Invitrogen, Lofer, Austria) by applying aliquots of 6.5 μl from each purification step and a dilution of 1 μl crude lysat in 10 μl loading-volume onto the gel. Proteins were mixed with 2.5 μl loading dye (Bio-Rad laboratories) and 1.5 μl Reducing agent (BioRad laboratories), denatured via 10 minutes at 70°C and loaded onto the gel, where 200 V were applied for 35 minutes.

MBD loaded bead preparation for DNA purification was performed as follows: Residual E.coli DNA was removed with buffer B (1.5 M NaCl, 20 mM HEPES, 20 mM β-Mercaptoethanol, 0.5 mM PMSF, 0.1 mM TritonX, pH = 8). The beads (100 μl) were then resuspended in 100 μl buffer A.

### MBD-loaded bead-based DNA Isolation

For DNA extraction using the MBD immobilized Ni-beads, 1 ml of serum was diluted with 1 ml of 2 × buffer A and incubated with an aliquot of 60 μl of prepared 50% MBD loaded bead suspension in buffer A for 2 h on a thermo mixer (Eppendorf, Hamburg, Germany) at 450 rpm at room temperature. After centrifugation at 1000 × g for 1 min beads were separated and used for DNA isolation. The supernatant was used for autoantibody-profiling (see below).

The MBD-loaded beads including the bound serum DNA were washed twice with buffer C (20 mM Hepes, 100 mM NaCl, 10% glycerol, pH = 8) and resuspended in 145 μl 10 mM Tris-Cl buffer, pH = 8. DNA was eluted from MBD loaded beads by ProteinaseK-digestion (20 mg/ml, Fermentas, St.Leon-Rot, Austria). The reaction contained 5 μl ProteinaseK in a volume of 150 μl and was incubated at 55°C for 20 min followed by an incubation at 65°C for 20 min. DNA was then isolated from the ProteinaseK reaction supernatant using Quiagen MinElute columns (Quiagen, Venlo, Netherlands). Purification was performed according to manufacturers instruction with an elution volume of 17 μl.

### Silica membrane-based DNA Isolation

DNA isolation from serum using common silica membrane-based isolation strategy was performed applying the Roche High pure template preparation kit (Roche Diagnostics, Mannheim, Germany). The protocol was adapted according to Müller HM. et al. [25]. Isolation was performed according to the manufacturer's instruction except the following protocol steps: the 800 μl of serum samples were split into 2 aliquots of 400 μl and each mixed with 400 μl of Roche Binding Buffer and 80 μl ProteinaseK (20 mg/μl Fermentas, St.Leon-Rot, Austria). After 15 min of incubation at 55°C, 200 μl isopropanol was added to each aliquot. Aliquots were mixed and an aliquot of 540 μl per serum sample were subsequently loaded four times to the column followed by a centrifugation step of 1 min at 8,000 × g. The flow through after each centrifugation was transferred back onto the same column and centrifuged again. Inhibitor-removal and washing steps were performed according to manufacturers' protocol, but the DNA was eluted in 55 μl of elution buffer.

### DNA quantification

DNA concentration was measured with the "Quant it" Pico Green kit (Invitrogen, Lofer, Austria) according to the manufacturers' protocol with a reduced reaction volume of 100 μl. Samples and standards were excited at 480 nm and emission was read at 520 nm using a BioRad IQ5 Real time PCR detection system. A five-point lambda DNA standard concentration curve ranging from 15 pg/μl to 250 pg/μl was measured in a volume of 100 μl.

### Microarray-based autoantibody tests

cDNA clones (fetal brain expression library) found reactive with serum from control individuals and breast cancer patients were used for protein expression and microarray generation (data not shown). Protein chips were processed as described by Stempfer R. et al. [26].

Serum samples, all originating from source 1, were either diluted 1 to 10 for untreated serum or 1 to 5 for samples after MBD-DNA isolation with PBST containing 3% nonfat dried bovine milk. Slides were blocked with DIGeasy Hyb (Roche Diagnostics, Mannheim, Germany) for 30 min, followed by two 5 min washing steps with PBST. A volume of 150 μl of diluted serum was applied onto each array and incubated for 2 h at room temperature. Slides were washed twice with PBST and detection of serum auto-antibodies was performed by incubation with Cy3 conjugated Affini Pure rabbit anti human IgA + IgG + IgM (Jackson Immuno Research) in a dilution of 1:25000 in PBST plus 3% nonfat dried bovine milk for 2 h followed by a 5 min washing step in PBST. Slides were scanned on a Gene Pix 4000a scanner (Axon Instruments) with a resolution of 10 μm at a photomultiplier tube setting of 700 PMT. Images were analyzed using Gene Pix software. Raw data were imported to GraphPad Prism (GraphPad Software, Inc.) to create Pearson correlation plots and to determine average signal intensities.

### DNA amplification and qPCR tests

DNA recovery of distinct isolation methods was controlled by two Multiplex-PCRs, which were performed with 6 primer pairs per reaction targeting the 5'-UTRs of CpG methylation controlled genes (primer sequences on request). An aliquot of 2 μl per serum DNA isolate served as template for the reaction and the (2-step)-PCR reaction setup and cycling was performed as published previously [27]. PCR products (10 μl) were loaded onto a 2% agarose gel containing 0.5 μg/ml ethidiumbromide.

qPCR analyses were carried out to assess the DNA integrity and enrichment of methylated DNA. All qPCR reactions were performed in a 384 well format on a Roche Light cycler 480 in reaction volumes of 10 μl containing 0.125 μM of each Primer, 0.3 U Hotstart Taq (Qiagen), 5% DMSO containing SYBR green to reach a dilution of 0.5 × in the final reaction and 166 μM dNTP-mix. A volume of 2 μl of Silica-membrane based DNA isolates served as template, whereas 1 μl template was used for the qPCR reaction of serum or plasma isolates, processed with MBD loaded beads. The PCR program was identical to the multiplex PCR program with the exception that for qPCR analyses 50 cycles were performed. Accuracy of Ct-values was assured via melting curve analyses of every analyzed reaction.

### Statistical tests

Mean and standard derivations were calculated for each data set. Pearson correlation coefficients were applied as a measurement of similarity between the data sets. A Wilcoxon rang sum test was applied for the not normally distributed data set of elevated DNA levels. None of the discussed data sets were normalized.

## Results

### Silica and MBD isolated DNA amount in serum/plasma of control individuals

To evaluate the new MBD loaded bead-based serum DNA isolation strategy 152 serum or plasma samples were processed with either a common silica-based DNA isolation workflow or with MBD loaded beads. Characterization of the serum DNA isolates showed concordant properties with respect to DNA recovery and relative amounts between patient groups, of both the MBD- and the optimized silica membrane- based DNA purification protocol.

**Figure 1:**
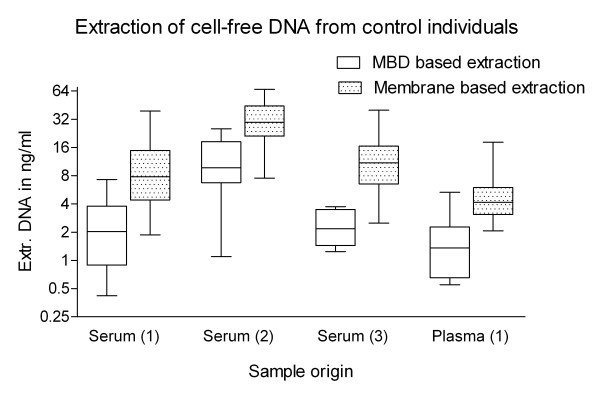
**Extraction of cell free DNA from control individuals**. Box plot of DNA amount isolated from 1 ml serum or plasma from (1) Austrian Institute of Technology (n = 12), (2) Austrian Red Cross (n = 24) and (3) AKH (n = 24). Serum DNA levels were dependent on serum source. Using the silica-based extraction protocol, mean amounts of DNA could be isolated ranging from (1), 11.9 ± 10.9 ng/ml (mean ± SD) and (3), 12.2 ± 9.7 ng/ml to (2), 39.7 ± 32.8 ng/ml. By contrast using the MBD-based protocol, serum DNA concentrations of (1) 2.5 ± 1.9 ng/ml, (2) 11.5 ± 7.3 ng/ml and (3) 2.4 ± 1 ng/ml were observed.

The mean DNA amount, isolated from 1 ml serum of 60 control individuals using the silica membrane based approach was 22.8 ± 25.7 ng/ml (mean ± SD.), ranging from 0.3 ng - 39.1 ng. By MBD-based isolation from 1 ml serum of 36 control individuals an average of 8.51 ± 7.3 ng/ml of DNA could be obtained, ranging from 0.7 ng - 25.4 ng. Extracted DNA amounts per ml serum (measured with Pico Green) varied dependent on the serum source, but showed similar trends when comparing both isolation strategies (Figure 1).

DNA extraction of plasma samples (source 1) was performed in triplicate in three independent experiments. A plasma DNA concentration of 1.8 ± 1.6 ng/ml (mean ± SD) was measured per ml plasma for the MBD protocol and 5.8 ± 5.1 ng/ml for the membrane based protocol (Figure 1). Comparing plasma DNA levels to DNA amounts isolated from 1 ml serum, we could ascertain that plasma DNA isolations of control individuals provide 57% (P = 0.002, Student t-test) of the DNA amount detected in serum of the same blood donors.

Selective DNA binding of MBD loaded beads: The MBD-isolated DNA yields were on average 25% (P < 0.001, Student t-test) of the DNA amounts obtained with the silica membrane based protocol. This reduced DNA amounts were observed in serum samples from sources 1-3 and in plasma samples from source 1 (Figure 1). However, this reduced DNA amounts isolated with MBD loaded beads did not affect the serum DNA recovery, because the agarose gel image based analyses of amplifiable serum DNA fragments was not influenced by the methylation status of DNA fragments.

To further elucidate the reason for the reduced DNA amount, which might be due to the selective binding character of the MBD protein for methylated DNA, qPCRs were performed on serum isolates targeting both known methylated and unmethylated gene regions. We made the observation that unmethylated DNA regions provided later Ct values when compared to silica membrane based isolation strategy but methylated DNA regions showed the same Ct values compared to DNA isolated with silica membrane (Additional File 1) These data indicated that the methylated DNA fraction is strongly enriched by the application of the MBD loaded bead extraction procedure. We concluded from this observation that the MBD DNA isolation procedure performs equal to silica membrane based DNA isolation strategies when methylated DNA is analyzed.

### DNA amounts in serum of patients with breast cancer

**Figure 2:**
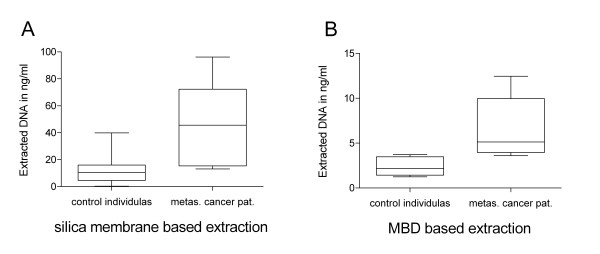
**Increased level of cell free DNA in serum of Breast cancer patients with metastasizing disease**. Amount of cell free DNA isolated from 1 ml serum of breast cancer patients with metastasizing tumors and control individuals obtained from AKH (source 3). An increased serum DNA amount was detected in sera from metastasizing tumors with both isolation strategies. (A), silica based isolation protocol (P = 0.0043, Wilcoxon test); (B) MBD loaded bead based purification (P = 0.0021, Wilcoxon test).

To get an impression whether data, produced with the MBD serum processing workflow, are comparable to published findings in current literature and our own experimental observation achieved with silica membrane based isolation strategy, DNA extractions with both strategies were performed on sera of breast cancer patients (Figure 2). The sera of six breast cancer patients with metastasizing disease and six control individuals were processed with MBD loaded beads. This was based on concentration measurement of serum DNA of 24 normal controls, 36 malign breast cancer patients, whereat 6 patients had a metastasizing disease, and 12 patients with a benign breast cancer applying a silica membrane based protocol (Table 1). The DNA amount in sera of malignant breast cancer patient without metastases was found to be at 13.7 ± 12.7 ng/ml. For patients with a benign disease we measured 12.6 ± 11.4 ng/ml. Both groups showed no significant difference compared to the control individuals which showed a concentration of 12.2 ± 7.9 ng/ml per ml serum (mean ± SD.). Including the measured serum DNA amounts of patients with metastasizing disease to the malign cancer group we observed a mean DNA concentration of 20.5 ± 21.4, which is an increase compared to non-cancer patients, but due to variation of the data not significant.

### Assessment of quality from cell free serum DNA

**Figure 3:**
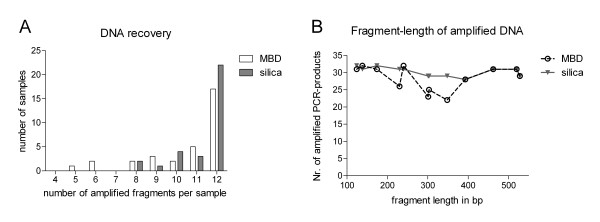
**Qualitiy of cell free serum DNA**. (A) reflects the amplification success for each sample using MBD or silica membrane based serum DNA. A maximum of 12 fragments per sample was possible and in sum 34 samples per isolation approach were analyzed. (B) shows the amplification success of each fragment getting amplified across all analyzed samples. Both plots (A, B) are based on the analysis of two multiplex PCRs performed on serum or plasma DNA isolates (source 1, 2).

To investigate the quality of DNA samples derived from the two isolation approaches we performed two independent multiplex-PCRs with six primers each, based on the hypothesis that the amplification success for different isolates might reflect the DNA quality. As described in Figure 3A, successful reactions with 12 amplified fragments were prevailing. Therefore, the isolated DNA was in general sufficient to perform two six-plex PCR, which demonstrated a good DNA quality. Amplification failure rate correlated with low isolated DNA amount, at which DNA levels below 1 ng per ml showed unsuccessful PCR reactions.

Although amplifying GC-rich templates, PCR products with a length from 125 bp up to 520 bp were amplified with high efficiencies in serum and in plasma samples (Figure 3B). Overall these observations show that although significantly smaller DNA amounts are isolated with the MBD based approach the method is still suitable to detect pathological changes in the patients like the increase of the DNA amount and the approach yields enough DNA to perform PCR reaction on the isolated methylated and unmethylated DNA fraction and subsequent gel image analyses with a comparable performance to the silica membrane based approach.

### Autoantibody tests of MBD processed serum

**Figure 4:**
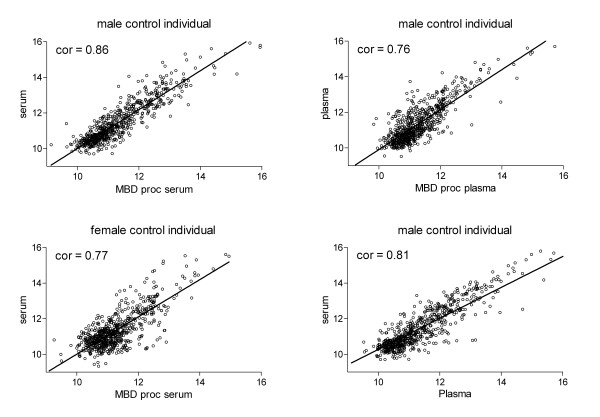
**Autoantibody tests of MBD processed serum**. Pearson correlation plots upon X-values of autoantibody protein micro arrays analyzing serum and plasma samples with and without MBD treatment. All samples originate from source 1 (AIT). Comparison of plasma and serum samples were performed on samples obtained from one single blood withdrawal. The cor-value states the Pearson's correlation.

To elucidate the effect of MBD based DNA isolation on the serum protein fraction, we compared auto-antibody signatures of native serum samples and samples after MBD-DNA isolation. Using an in house fabricated protein array comprising 642 different proteins [26], MBD treated serum/plasma and untreated serum/plasma from six males and females in duplicate were analyzed concerning their autoantibody profile. Array data showed a Pearson correlation form 0.67 to 0.86 for the comparison of MBD treated serum/plasma and untreated serum/plasma, whereof two representative samples were plotted in Figure 4. The log 2 value of the median array signal intensities of all analyzed MBD processed serum samples was 11.2 ranging from 9.7 to 12.9 in comparison to 10.8 for untreated serum or plasma (range 10.2 to 12.9), excluding an experimental bias introduced by the MBD loaded bead DNA isolation process. Also patient gender or blood treatment (serum/plasma) did not affect median signal intensities significantly. These data suggest that MBD isolation has a minor effect on the autoantibody pattern of different sera and plasma samples, but rather retains protein function. Correlation plots (Figure 4) furthermore indicate same patterns of autoantibodies for serum and plasma samples.

## Discussion

In serum or plasma of healthy adults as well as breast cancer patients the amount of cell free DNA [28] is low, but of great interest for minimal invasive diagnostics [29]. We developed a serum processing workflow, which enables to perform DNA studies followed by autoantibody tests out of the same serum sample. This addresses the problem of limited access to clinical sample material, which is a constraint of many researchers and clinicians [30].

In this present study we could show that it is possible to isolate serum DNA, suitable for PCR analyses and especially methylation analyses of cell free DNA, using a sample processing based on the affinity of MBD to methylated DNA [16]. Furthermore, the gentle sample treatment in the DNA isolation step holds the possibility of additional proteomic analyses and miRNA isolations or autoantibody tests (the latest we performed in this work here). We present a new application of the MBD protein using an advanced bead based approach to isolate DNA from serum and plasma samples thereby retaining protein function. For this we developed a simplified protein purification procedure (Additional File 2).

The expectation of small DNA amounts in serum was encountered by our strategy to replace the standard salt elution by a ProteinaseK digestion of the MBD protein to regain the entire MBD bound DNA. This allowed us to isolate 2.4 ng/ml DNA out of serum of control individuals and 6.6 ng/ml of metastasizing breast cancer patients. However, DNA amounts detected with a membrane based isolation procedure were on average about three times higher due to the relative low affinity of the MBD to unmethylated DNA (Additional File 1).

This attribute of the MBD protein derived from MeCP2 to bind predominantly methylated DNA, is due to its actual biological function as transcriptional regulator [31,32]. At qPCR based methylation studies it turned out, that there is a certain amount of unmethylated DNA bound by the MBD protein even at salt concentrations above 0.6 M usually recommended for elution of methylated DNA from the protein (data not shown). Although there was a significant enrichment of methylated CpG-rich fragments in the DNA fraction after MBD-treatment, we propose to include an additional methyl-sensitive restriction digestion or sodium bisulfite treatment to guarantee accurate methylation studies based on the MBD protein, which is also suggested by other researchers in concordant literature [16,22].

In this study we further investigated if the MBD loaded bead workflow is suitable for plasma DNA isolations. This approach enabled us to highlight another topic, namely the direct comparison of DNA levels in plasma and serum, upon paralleled preparation of plasma and serum samples out of a single blood withdrawal. We found 5.8 ± 5.2 ng/ml of cell free DNA in plasma of control individuals versus 11.9 ± 10.9 ng/ml prevailing in 1 ml serum of the same patients. The same ratio of DNA amounts was observed using the MBD loaded bead isolation protocol. There are reports that higher DNA levels in serum are due to the clotting process and the associated release of DNA from destroyed white blood cells [3,33] and therefore do not contribute to alterations of cell-free DNA, tested in different diagnostic assays [34]. The fact of additional normal DNA derived from blood lymphocytes detected in serum may also be responsible for sample source dependant varying DNA amounts observed in our study.

Another indication for the universal applicability of the MBD DNA isolation protocol was the affirmation of the widely discussed increase of cell free serum DNA levels in breast cancer patients [35,36] with both purification approaches, although this elevated DNA amounts could be observed in few patient sera with a non-metastasis malign neoplasm and not in sera of benign tumors. Therefore our data might rather agree with observations of metastasis cancerous diseases [37] than with an increase of cell free DNA of overall malignant cancers [11].

Because autoantibody-based serum analyses is of high interest for minimal invasive diagnostics, and research efforts are on the way for elucidation of antigenic biomarkers, paralleled analysis of the protein fraction in addition to DNA based methods are of high relevance for efficient usage of limiting amounts of retrospective clinical samples. Serum-autoantibody tests hold the potential to recognize array bound peptides differentially dependent on whether these antibodies were produced in sera of control individuals or patients with a cancerous disease. These peptide sequences may serve as useful biomarkers and soon could support clinical diagnoses [38,39].

## Conclusions

In this study we performed first tests with this new bead based serum processing workflow, where we primarily used the DNA-binding characteristics of the MBD protein to isolate DNA from sera and plasma of control individuals and breast cancer patients. Hence the MBD based DNA isolation holds the potential to combine DNA methylation studies with the detection of any serum protein marker applicable for clinical diagnoses. Thus this procedure offers efficient usage of the limiting amounts of clinical samples for combined testing of DNA and proteins. This combination of DNA based diagnostic tests and the analysis of the erum/plasma protein fraction should be an important step towards non-invasive cancer tests, because of the increased statistical power gained by two independent methods. This would enhance diagnostic reliability and accuracy.

## Competing interests

The authors declare that they have no competing interests.

## Authors' contributions

MW carried out PCR, qPCR, purifications and data analyses and drafted the manuscript. WP performed the gel image analyses. JP participated statistical analyses and plotted the data. MH participated at the expression of the protein in E.coli. CFR collected the information about breast cancer patients. CS collected a patient cohort participated in the collection of clinical data and contributed to the manuscript. CJ collected a patient cohort and contributed to the manuscript. CN participated in design and coordination of the study. AW conceived the study and participated in its design and contributed to manuscript. All authors read and approved the final paper.

## Pre-publication history

The pre-publication history for this paper can be accessed here:

http://www.biomedcentral.com/1472-6890/11/11/prepub

## Supplementary Material

Additional file 1 **Differential methylated DNA fragments**. Ct-values of differential methylated DNA fragments: DNA was isolated from serum (source 1, n = 8) either using the silica membrane based approach or the MBD approach. Four different 5'-UTR regions with known methylation status in healthy peripheral blood were tested, SALL3, ESR1, CHFR (all unmethylated loci) and ZNF502 (biallelic methylated loci). A mean difference between Ct-values regarding the three unmethylated genes of 2.2 was observed; highlighting the reduced amounts of unmethylated DNA, isolated by MBD loaded bead approach. For the ZNF502 gene locus, which is methylated in healthy adults, no significant difference between Ct-values of two isolation approaches was detected. Click here for file

Additional file 2 **SDS gel of MBD purification**. The Coomassie stained SDS gel outlines our simplified MBD purification protocol and enabled us to control correct MBD-bead assembly. Aliquots of the several purification steps were loaded onto the gel. Lane 1, 5 kDa Page Ruler; Lane 2, crude lysate (1:5 dilution); Lane 3, supernatant of the binding reaction; Lane 4 and 5, subsequent washing steps with buffer A containing 10 mM imidazol; Lane 6, purified MBD protein eluted from Ni-NTA beads, where one band at 11 kDa remained. Click here for file

## References

[B1] Lo YM Chiu RW Next-generation sequencing of plasma/serum DNA: an emerging research and molecular diagnostic tool Clin Chem 2009 55 607 608 10.1373/clinchem.2009.123661 19233905

[B2] Dobrzycka B Terlikowski SJ Mazurek A Kowalczuk O Niklinska W Chyczewski L Kulikowski M Circulating free DNA, p53 antibody and mutations of KRAS gene in endometrial cancer Int J Cancer 2009 10.1002/ijc.25077 19960433

[B3] Fleischhacker M Schmidt B Circulating nucleic acids (CNAs) and cancer--a survey Biochim Biophys Acta 2007 1775 181 232 17137717 10.1016/j.bbcan.2006.10.001

[B4] Anker P Mulcahy H Chen XQ Stroun M Detection of circulating tumour DNA in the blood (plasma/serum) of cancer patients Cancer Metastasis Rev 1999 18 65 73 10.1023/A:1006260319913 10505546

[B5] Wang BG Huang HY Chen YC Bristow RE Kassauei K Cheng CC Roden R Sokoll LJ Chan DW Shih I Increased plasma DNA integrity in cancer patients Cancer Res 2003 63 3966 3968 12873992

[B6] Elrick MM Walgren JL Mitchell MD Thompson DC Proteomics: recent applications and new technologies Basic Clin Pharmacol Toxicol 2006 98 432 441 10.1111/j.1742-7843.2006.pto_391.x 16635100

[B7] Anker P Mulcahy H Stroun M Circulating nucleic acids in plasma and serum as a noninvasive investigation for cancer: time for large-scale clinical studies? Int J Cancer 2003 103 149 152 10.1002/ijc.10791 12455027

[B8] Schaub NP Jones KJ Nyalwidhe JO Cazares LH Karbassi ID Semmes OJ Feliberti EC Perry RR Drake RR Serum proteomic biomarker discovery reflective of stage and obesity in breast cancer patients J Am Coll Surg 2009 208 970 978 10.1016/j.jamcollsurg.2008.12.024 19476873

[B9] Kijanka G Murphy D Protein arrays as tools for serum autoantibody marker discovery in cancer J Proteomics 2009 72 936 944 10.1016/j.jprot.2009.02.006 19258055

[B10] Anderson KS Wong J Vitonis A Crum CP Sluss PM Labaer J Cramer D p53 autoantibodies as potential detection and prognostic biomarkers in serous ovarian cancer Cancer Epidemiol Biomarkers Prev 2010 19 859 868 10.1158/1055-9965.EPI-09-0880 20200435 PMC2838192

[B11] Wu TL Zhang D Chia JH Tsao KH Sun CF Wu JT Cell-free DNA: measurement in various carcinomas and establishment of normal reference range Clin Chim Acta 2002 321 77 87 10.1016/S0009-8981(02)00091-8 12031596

[B12] Seefeld M El TS Fan AX Hahn S Holzgreve W Zhong XY Parallel assessment of circulatory cell-free DNA by PCR and nucleosomes by ELISA in breast tumors Int J Biol Markers 2008 23 69 73 18629778 10.1177/172460080802300202

[B13] Lewis JD Meehan RR Henzel WJ Maurer-Fogy I Jeppesen P Klein F Bird A Purification, sequence, and cellular localization of a novel chromosomal protein that binds to methylated DNA Cell 1992 69 905 914 10.1016/0092-8674(92)90610-O 1606614

[B14] Jones PL Veenstra GJ Wade PA Vermaak D Kass SU Landsberger N Strouboulis J Wolffe AP Methylated DNA and MeCP2 recruit histone deacetylase to repress transcription Nat Genet 1998 19 187 191 10.1038/561 9620779

[B15] Weitzel JM Buhrmester H Stratling WH Chicken MAR-binding protein ARBP is homologous to rat methyl-CpG-binding protein MeCP2 Mol Cell Biol 1997 17 5656 5666 9271441 10.1128/mcb.17.9.5656 PMC232414

[B16] Klose RJ Sarraf SA Schmiedeberg L McDermott SM Stancheva I Bird AP DNA binding selectivity of MeCP2 due to a requirement for A/T sequences adjacent to methyl-CpG Mol Cell 2005 19 667 678 10.1016/j.molcel.2005.07.021 16137622

[B17] Cross SH Charlton JA Nan X Bird AP Purification of CpG islands using a methylated DNA binding column Nat Genet 1994 6 236 244 10.1038/ng0394-236 8012384

[B18] Shiraishi M Chuu YH Sekiya T Isolation of DNA fragments associated with methylated CpG islands in human adenocarcinomas of the lung using a methylated DNA binding column and denaturing gradient gel electrophoresis Proc Natl Acad Sci USA 1999 96 2913 2918 10.1073/pnas.96.6.2913 10077611 PMC15869

[B19] Serre D Lee BH Ting AH MBD-isolated Genome Sequencing provides a high-throughput and comprehensive survey of DNA methylation in the human genome Nucleic Acids Res 2010 38 391 399 10.1093/nar/gkp992 19906696 PMC2811030

[B20] Zou H Harrington J Rego RL Ahlquist DA A novel method to capture methylated human DNA from stool: implications for colorectal cancer screening Clin Chem 2007 53 1646 1651 10.1373/clinchem.2007.086223 17712002

[B21] Yu Y Blair S Gillespie D Jensen R Myszka D Badran AH Ghosh I Chagovetz A Direct DNA methylation profiling using methyl binding domain proteins Anal Chem 2010 82 5012 5019 10.1021/ac1010316 20507169 PMC2946844

[B22] Yegnasubramanian S Lin X Haffner MC DeMarzo AM Nelson WG Combination of methylated-DNA precipitation and methylation-sensitive restriction enzymes (COMPARE-MS) for the rapid, sensitive and quantitative detection of DNA methylation Nucleic Acids Res 2006 34 e19 10.1093/nar/gnj022 16473842 PMC1363782

[B23] Zanetti-Dallenbach R Wight E Fan AX Lapaire O Hahn S Holzgreve W Zhong XY Positive correlation of cell-free DNA in plasma/serum in patients with malignant and benign breast disease Anticancer Res 2008 28 921 925 18507037

[B24] Cross SH Charlton JA Nan X Bird AP Purification of CpG islands using a methylated DNA binding column Nat Genet 1994 6 236 244 10.1038/ng0394-236 8012384

[B25] Muller HM Widschwendter A Fiegl H Ivarsson L Goebel G Perkmann E Marth C Widschwendter M DNA methylation in serum of breast cancer patients: an independent prognostic marker Cancer Res 2003 63 7641 7645 14633683

[B26] Simon R Lam A Li MC Ngan M Menenzes S Zhao Y Analysis of Gene Expression Data Using BRB-Array Tools Cancer Inform 2007 3 11 17 19455231 PMC2675854

[B27] Weinhaeusel A Thiele S Hofner M Hiort O Noehammer C PCR-based analysis of differentially methylated regions of GNAS enables convenient diagnostic testing of pseudohypoparathyroidism type Ib Clin Chem 2008 54 1537 1545 10.1373/clinchem.2008.104216 18617581

[B28] Zhong XY Hahn S Kiefer V Holzgreve W Is the quantity of circulatory cell-free DNA in human plasma and serum samples associated with gender, age and frequency of blood donations? Ann Hematol 2007 86 139 143 17024502 10.1007/s00277-006-0182-5

[B29] Gahan PB Swaminathan R Circulating nucleic acids in plasma and serum. Recent developments Ann N Y Acad Sci 2008 1137 1 6 10.1196/annals.1448.050 18837917

[B30] Nygaard V Hovig E Options available for profiling small samples: a review of sample amplification technology when combined with microarray profiling Nucleic Acids Res 2006 34 996 1014 10.1093/nar/gkj499 16473852 PMC1363777

[B31] Ho KL McNae IW Schmiedeberg L Klose RJ Bird AP Walkinshaw MD MeCP2 binding to DNA depends upon hydration at methyl-CpG Mol Cell 2008 29 525 531 10.1016/j.molcel.2007.12.028 18313390

[B32] Jang JS Lee SJ Choi JE Cha SI Lee EB Park TI Kim CH Lee WK Kam S Choi JY Kang YM Park RW Kim IS Cho YL Jung TH Han SB Park JY Methyl-CpG binding domain 1 gene polymorphisms and risk of primary lung cancer Cancer Epidemiol Biomarkers Prev 2005 14 2474 2480 10.1158/1055-9965.EPI-05-0423 16284366

[B33] Holdenrieder S Stieber P Chan LY Geiger S Kremer A Nagel D Lo YM Cell-free DNA in serum and plasma: comparison of ELISA and quantitative PCR Clin Chem 2005 51 1544 1546 10.1373/clinchem.2005.049320 16040855

[B34] Andriani F Conte D Mastrangelo T Leon M Ratcliffe C Roz L Pelosi G Goldstraw P Sozzi G Pastorino U Detecting lung cancer in plasma with the use of multiple genetic markers Int J Cancer 2004 108 91 96 10.1002/ijc.11510 14618621

[B35] Kohler C Radpour R Barekati Z Asadollahi R Bitzer J Wight E Burki N Diesch C Holzgreve W Zhong XY Levels of plasma circulating cell free nuclear and mitochondrial DNA as potential biomarkers for breast tumors Mol Cancer 2009 8 105 10.1186/1476-4598-8-105 19922604 PMC2780981

[B36] Van dA Elst HJ Van Laere SJ Maes H Huget P van DP Van Marck EA Vermeulen PB Dirix LY The presence of circulating total DNA and methylated genes is associated with circulating tumour cells in blood from breast cancer patients Br J Cancer 2009 100 1277 1286 10.1038/sj.bjc.6605013 19367284 PMC2676551

[B37] Tokuhisa Y Iizuka N Sakaida I Moribe T Fujita N Miura T Tamatsukuri S Ishitsuka H Uchida K Terai S Sakamoto K Tamesa T Oka M Circulating cell-free DNA as a predictive marker for distant metastasis of hepatitis C virus-related hepatocellular carcinoma Br J Cancer 2007 97 1399 1403 10.1038/sj.bjc.6604034 17940509 PMC2360234

[B38] Ionov Y A high throughput method for identifying personalized tumor-associated antigens Oncotarget 2010 1 148 155 20711419 10.18632/oncotarget.118 PMC2920534

[B39] Ludwig N Keller A Comtesse N Rheinheimer S Pallasch C Fischer U Fassbender K Steudel WI Lenhof HP Meese E Pattern of serum autoantibodies allows accurate distinction between a tumor and pathologies of the same organ Clin Cancer Res 2008 14 4767 4774 10.1158/1078-0432.CCR-07-4715 18676746

