# Supplementary figures and images for: Methyl-binding domain protein-based DNA isolation from human blood serum combines DNA analyses and serum-autoantibody testing
Source: BMC Clin Pathol. 2011 Sep 6;11:11. doi: 10.1186/1472-6890-11-11 (PMC3180258; doi:10.1186/1472-6890-11-11)

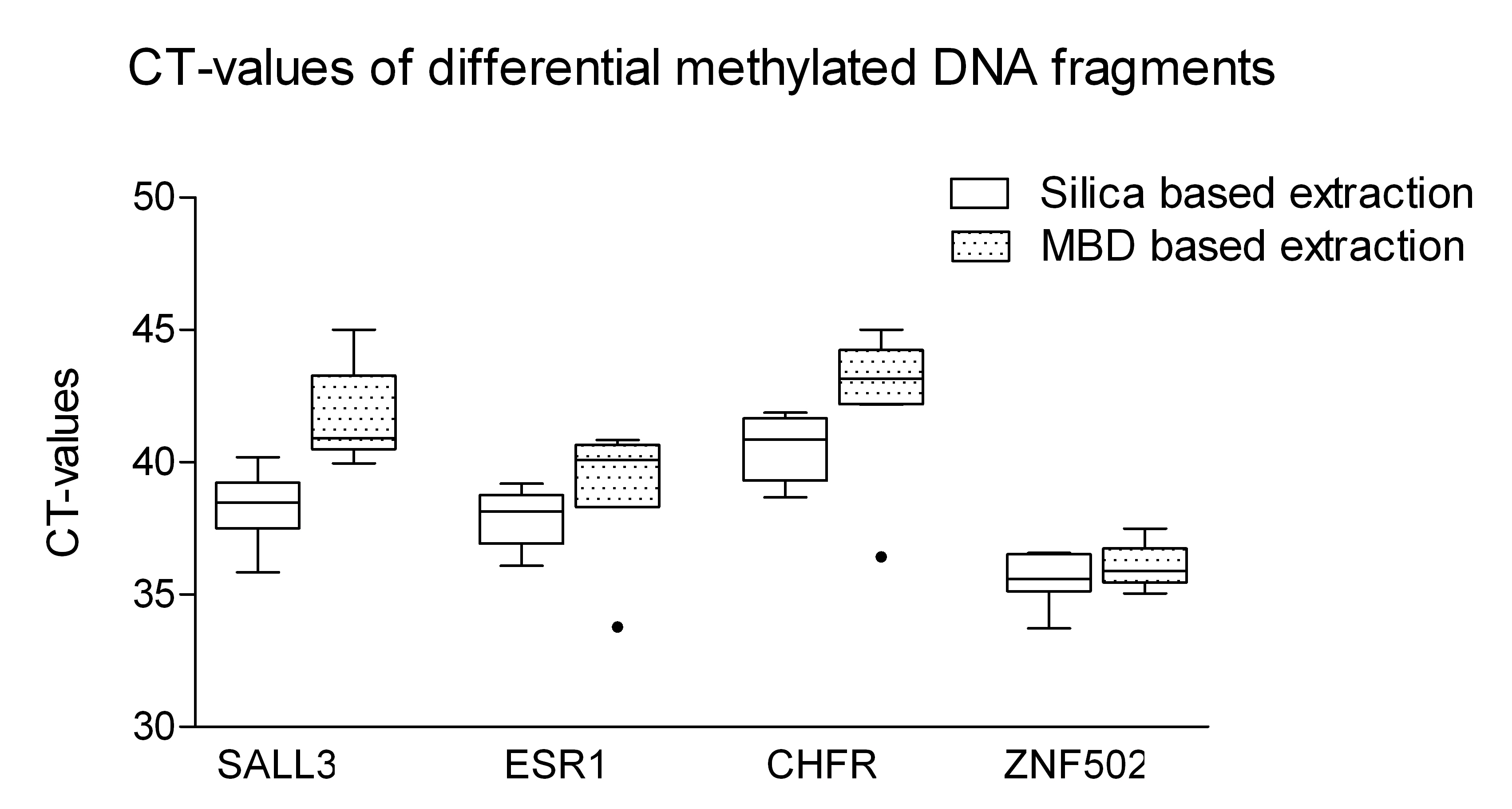

Supplement: Additional file 1 — Differential methylated DNA fragments. Ct-values of differential methylated DNA fragments: DNA was isolated from serum (source 1, n = 8) either using the silica membrane based approach or the MBD approach. Four different 5'-UTR regions with known methylation status in healthy peripheral blood were tested, SALL3, ESR1, CHFR (all unmethylated loci) and ZNF502 (biallelic methylated loci). A mean difference between Ct-values regarding the three unmethylated genes of 2.2 was observed; highlighting the reduced amounts of unmethylated DNA, isolated by MBD loaded bead approach. For the ZNF502 gene locus, which is methylated in healthy adults, no significant difference between Ct-values of two isolation approaches was detected. [file 1472-6890-11-11-S1.JPEG]

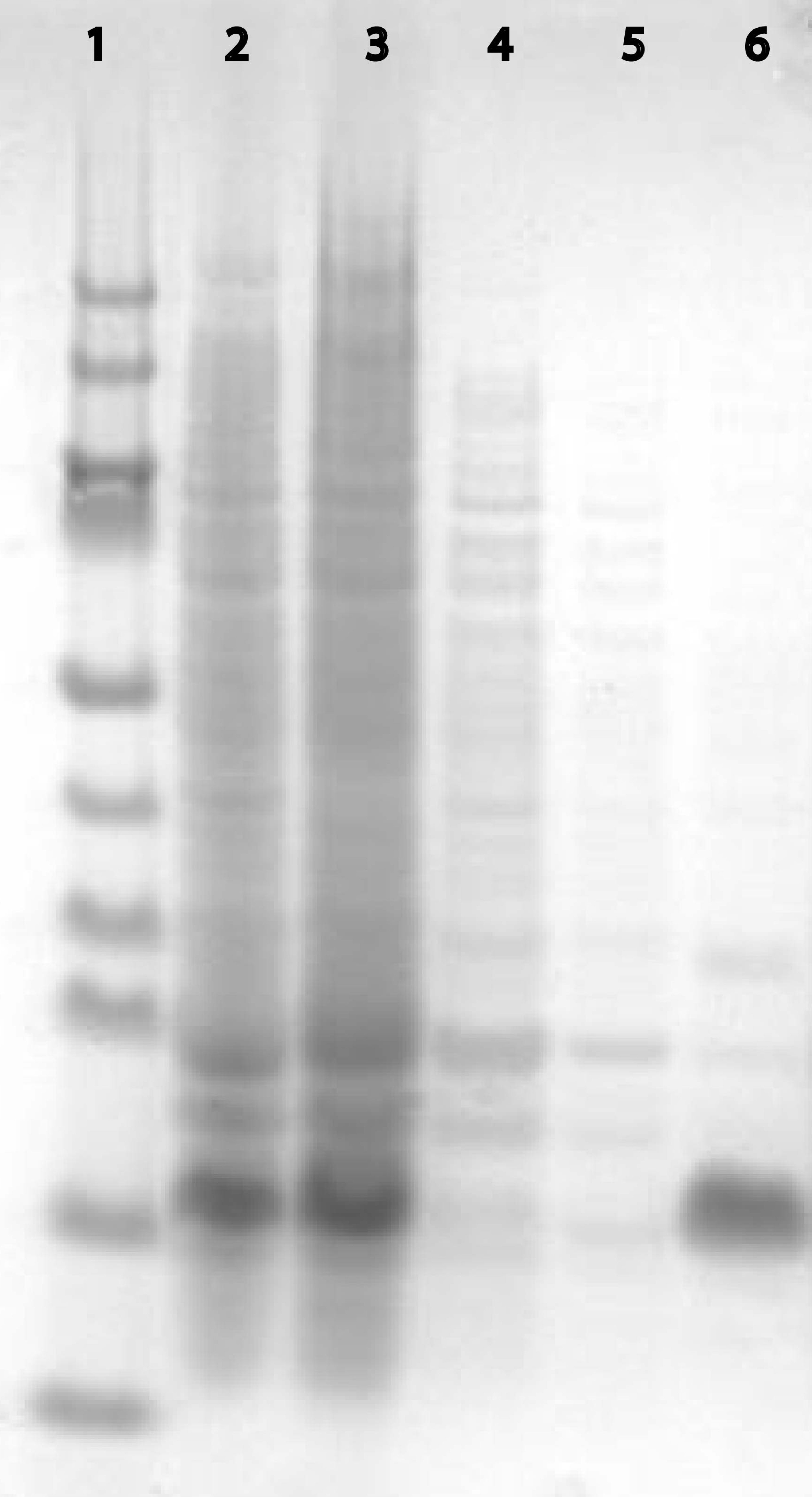

Supplement: Additional file 2 — SDS gel of MBD purification. The Coomassie stained SDS gel outlines our simplified MBD purification protocol and enabled us to control correct MBD-bead assembly. Aliquots of the several purification steps were loaded onto the gel. Lane 1, 5 kDa Page Ruler; Lane 2, crude lysate (1:5 dilution); Lane 3, supernatant of the binding reaction; Lane 4 and 5, subsequent washing steps with buffer A containing 10 mM imidazol; Lane 6, purified MBD protein eluted from Ni-NTA beads, where one band at 11 kDa remained. [file 1472-6890-11-11-S2.JPEG]
